# Arterial Spin Labeling (ASL) fMRI: Advantages, Theoretical Constrains and Experimental Challenges in Neurosciences

**DOI:** 10.1155/2012/818456

**Published:** 2012-02-22

**Authors:** Ajna Borogovac, Iris Asllani

**Affiliations:** Department of Radiology, Columbia University, New York, NY 10032, USA

## Abstract

Cerebral blood flow (CBF) is a well-established correlate of brain function and therefore an essential parameter for studying the brain at both normal and diseased states. Arterial spin labeling (ASL) is a noninvasive fMRI technique that uses arterial water as an endogenous tracer to measure CBF. ASL provides reliable absolute quantification of CBF with higher spatial and temporal resolution than other techniques. And yet, the routine application of ASL has been somewhat limited. In this review, we start by highlighting theoretical complexities and technical challenges of ASL fMRI for basic and clinical research. While underscoring the main advantages of ASL versus other techniques such as BOLD, we also expound on inherent challenges and confounds in ASL perfusion imaging. In closing, we expound on several exciting developments in the field that we believe will make ASL reach its full potential in neuroscience research.

## 1. Introduction

Until we find a method that can directly and noninvasively measure the production and consumption of ATP, we must rely on measuring physiological correlates of ATP to study the brain at various functional states such as activation and disease. Cerebral blood flow (CBF) is one such correlate. CBF delivers glucose and oxygen to the brain to maintain basal ATP production and to replenish it during increased neuronal activity. Changes in CBF are concomitant with changes in neuronal activity, such as those occurring during task activation, or changes in metabolism that often indicate presence of disease [1]. Because of this close coupling with brain function, CBF is an essential physiological parameter, which is why much effort has been dedicated to developing reliable methods for measuring it.

All the major methods that have been developed for measuring CBF are based on the principles of compartmental modeling and tracer kinetics. These principles yield models that describe the dynamics of a tracer as it crosses the arterial tree into the brain's microvasculature (nondiffusible tracers) and into the tissue (diffusible tracers) prior to venous washout. Different methods use different types of tracers. One of the main advantages of ASL fMRI is that, unlike most other methods, it uses arterial water as an endogenous tracer and thus does not require injection of exogenous tracers that can be uncomfortable and potentially harmful. Also, because ASL is noninvasive, it is safe to repeat over time and can therefore be used to track changes in CBF such as those due to disease progression or drug therapy. Important, ASL yields an absolute measurement of CBF and therefore any change in flow can be expressed in physiologically meaningful units rather than as a % change. Furthermore, ASL yields CBF images with higher spatial and temporal resolution than any other current technique.

And yet, despite these numerous advantages, ASL has yet to emerge as the technique of choice for measuring CBF at baseline or during task activation. The main motivation for this review was to postulate on the main reasons for this relatively slow-paced advancement of ASL. To this aim, we first start with a description of the general theoretical framework of ASL including various labeling implementations that have been developed to date. Then we highlight the key advantages of ASL versus other methods while expounding on its theoretical limitations and experimental challenges. We close with a review of recent developments in the field that have the promise of making ASL realize its full potential in brain research.

## 2. Theoretical Framework of ASL fMRI

**Figure 1:**
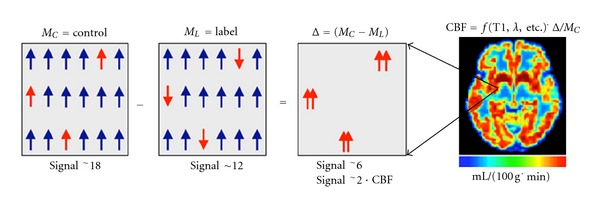
Schematic presentation of how ASL signal is obtained. The first three panels represent the signal from a single imaged voxel that originates from the control (left), label (center), and control-label difference (right) panels, respectively. The numbers are not meant to represent real flow. A real CBF image is shown in the rightmost panel. The color bar represents flow in [0–107] mL/100 g·min range. Note that, as mentioned in text, the difference ∆ = (*M*_*C*_ − *M*_*L*_) image is converted to a single CBF image via a function that includes physiological and MR parameters such as relaxation rates, transit times, and blood tissue water partition coefficient, *λ*.

In all ASL methods, the proton spins of the arterial water are labeled prior to reaching the imaged volume. “Labeling” refers to a change in the magnetic state of the inflowing spins by either saturation or inversion. Once the spins have been labeled, and after a time delay that allows for them to exchange with the tissue, an image, referred to as “labeled,” is acquired [2]. In this image, the blood water is in a different magnetization state from that of the static tissue water. If one models the static tissue as an upward, +1, vector then the labeled water is either 0 (saturation) or −1 (inversion). The signal from a given voxel in the labeled image represents a sum over both blood and tissue spins (Figure 1, left panel).

 In addition to the “labeled” image, a “control” image is acquired in which, ideally, the arterial water spins have not been altered, and therefore both the static tissue spins and blood spins are in the same magnetic state at acquisition (Figure 1, middle panel). Now one can see (Figure 1, right panel) that at each imaged voxel, the difference between the control and labeled images is proportional to the amount of flow supplying that voxel.

The ASL signal is typically expressed as a fractional ratio (1) between the difference, (*M*_*C*_ − *M*_*L*_), image and the control, *M*_*C*_, image, which represents the equilibrium magnetization [4]

(1) $$\text{AS}{\text{L}}_{\text{signal}}=\frac{\left(M_{C}-M_{L}\right)}{M_{C}}.$$

A CBF image is computed by applying a set of measured or assumed physiological and MR parameters on the ASL-signal image to obtain voxelwise flow values in absolute physiological units of flow (Figure 1). Multiple pairs of labeled and control images are acquired to ensure that an average of several cardiac outputs has been obtained.

Since the original innovation of the basic ASL technique by Williams et al. in 1992 [4], numerous ASL sequences have been developed. Based on how labeling is achieved, these sequences are commonly categorized as either continuous ASL (CASL), where labeling duration is relatively long (seconds), or pulsed ASL (PASL), where short (milliseconds) labeling pulses are used [5]. Recently, ASL sequences have been modified to include background suppression pulses that aim at suppressing the static tissue signal in order to improve the stability and SNR of the ASL [6, 7]. Thus, several permutations exist even within the same major ASL category. Moreover, recent developments such as pseudo-continuous ASL (pCASL) [8] and velocity selective ASL (VSASL) [9] have made this dichotomization into CASL and PASL somewhat obsolete. While recognizing the limitations of categorizations in general, in this review we highlight the basic principles as well as advantages and limitations of CASL, pCASL, PASL, and VSASL, separately.

### 2.1. Continuous Arterial Spin Labeling (CASL)

In CASL, the inflowing arterial water spins are “continuously” labeled via inversion through a process called adiabatic fast passage (AFP) [2, 4, 10, 11]. For adiabatic inversion to occur, two conditions must be met: first, the entire labeling process needs to be faster than the relaxation times (hence the term “fast” in AFP), and, second, the orientation of the effective magnetic field, *B*_eff_, needs to change at sufficiently slow rate so that the angle between *B*_eff_ and the net magnetization remains constant (hence the term “adiabatic”). Theoretically, the labeling pulse must be long enough for the steady state to be reached [4]. However, taking into consideration hardware and experimental restrictions, the labeling pulse is typically ~2 s. The inversion occurs at a thin plane referred to as the “labeling plane” generally positioned in the carotids where the average flow velocity ensures that both adiabatic conditions are met.

One of the major drawbacks of CASL is the requirement for a long labeling pulse to bring about the adiabatic inversion. This requirement complicates CASL on both theoretical and practical standpoints. Theoretically, long off-resonance RF pulses cause signal loss due to what are commonly referred to as magnetization transfer (MT) effects [12]. Because the control images do not require labeling pulses, their signal would not be affected by MT. Consequently, the difference (*M*_*C*_ − *M*_*L*_) would reflect not only the blood flow but also the loss in signal due to MT effects that are present in *M*_*L*_ but not *M*_*C*_. The MT effects constrained the initial application of CASL to a single slice where in order to balance these effects, two labeled images were acquired, one above and one below the imaged slice [13]. To circumvent this constraint, Alsop and Detre applied an amplitude-modulated (AM) RF pulse with the same duration as the labeling pulse during the acquisition of the control image [14]. While causing approximately the same MT effects during the acquisition of the control images as during the labeled, the AM pulses affect a double inversion, that is, no net inversion, on the inflowing spins thus meeting the requirement of no change in magnetization state during the acquisition of the control image [14]. The introduction of the AM pulses moved CASL from single slice to whole-brain imaging thus opening new venues for its applications.

Practically, the requirement for long RF pulses is so taxing on the hardware that most commercially available MR scanners do not offer CASL as part of their software package. Several techniques have been developed to overcome this problem. They differ in the way label and control scans are realized [15]. In a recent study, Pohmann et al. investigated the sensitivity of four of these CASL techniques using both simulation and experimental data [15]. Briefly, the CASL implementations tested were the following:

- dual-coil (DC-CASL) which works as the conventional CASL method described above except that it uses two separate coils for labeling and imaging, respectively [16, 17]. Since labeling is achieved independently from imaging, the MT effects are completely avoided. However, the need for an additional transmit channel increases the level of technical expertise and hardware requirements therefore making DC-CASL difficult for routine application.
- Almost continuous ASL (ACASL) where the labeling pulse is regularly and briefly interrupted thus alleviating the burden on the RF amplifier to produce long pulses. To ensure equal MT effects on both label and control images, two different variations of the control acquisition were considered: one restricted the imaged volume to a single slice, referred to as ss-ACASL, whereas the other allowed for multislice acquisition hence referred to as ms-ACASL.
- Pseudo-continuous ASL (pCASL) which instead of a long rectangular labeling pulse applies short and shaped pulses in combination with rephrased gradients to adiabatically invert the inflowing spins [18]. An advantage of this sequence compared to DC-CASL is that it does not require an additional labeling coil and it can be implemented with the standard coils provided by the manufacturer.

As expected from theoretical considerations, ss-ACASL yielded higher SNR followed by the dual-coil DC-CASL acquisition. However, both these methods limit the application of CASL for routine CBF measurement as the first is constrained to a single slice whereas the second requires additional personnel expertise and hardware work to be implemented. Realistically, pCASL is the optimum choice because it can be widely applied using standard hardware without substantial sacrifice in SNR. As discussed below, we believe that currently pCASL holds the best promise for routine application of ASL at higher magnetic fields.

### 2.2. Pseudo-Continuous ASL (pCASL)

As mentioned above, one of the major drawbacks of CASL is the requirement for long RF labeling pulses, which, in addition to causing MT effects that confound the ASL signal, are also quite taxing on the hardware and therefore not widely available. Because PASL uses short RF pulses, it is less susceptible to MT effects. However, PASL suffers from low sensitivity compared to CASL; the SNR of PASL can be 30% to 50% lower than that of CASL [19–21].

Pseudo-continuous ASL was developed as an intermediate technique that takes advantage of CASL's superior SNR and PASL's high labeling efficiency without the need for long labeling pulses [18, 20, 22]. This is achieved by using a train of short RF pulses rather than continuous RF to bring about inversion of the water spins [22]. Given that the ASL signal represents the amount of labeled spins captured within an imaged voxel, a more efficient labeling pulse and decreased MT effects in pCASL should be reflected in higher SNR compared to conventional ASL. This theoretical statement was empirically evaluated by Wu et al. who compared optimized pCASL with standard PASL and CASL at 3T [20]. The authors found that pCASL provides a 50% improvement in SNR compared to PASL and an 18% increase in labeling efficiency compared to CASL (80% versus 68%, resp.) [20].

In addition to increased intrasubject SNR and feasibility, pCASL has been shown to have lower intersubject variability compared to standard ASL [23]. Gevers et al. compared the reproducibility and reliability of pCASL, conventional CASL, and PASL based on images acquired on 6 healthy volunteers who were scanned twice at three different imaging centers [23]. When compared to conventional CASL and PASL, pCASL with background suppression showed the least data dispersion and best reproducibility [23]. While Gevers et al. study was done on only healthy young volunteers, Xu et al. compared the reliability and precision of pCASL with those of ^15^O PET on 8 young healthy subjects and 14 elderly, 2 of which were diagnosed with early Alzheimer's disease (AD) [24]. The authors found that compared to previous ASL and PET perfusion studies, pCASL offers as good or even better reliability in repeated measurements for both young and elderly subjects. The relationship between quantitative ASL CBF, age, and AD was found consistent with previous reports, further validating the approach [24].

Recently, Dai et al. have proposed a new approach for achieving pseudo-continuous labeling of the arterial spins with higher efficiency than CASL for the same RF power deposition at 3T [18]. The main new feature of this implementation is that it replaces the rectangular train of RF pulses with a more sophisticated Hanning pulse to achieve a more precise labeling profile without compromising the labeling efficiency of the previous pCASL implementations [18]. In good agreement with the Wu study, labeling efficiency for the in vivo imaging with pCASL was 81% for the same power as standard CASL at 3T.

**Figure 2:**
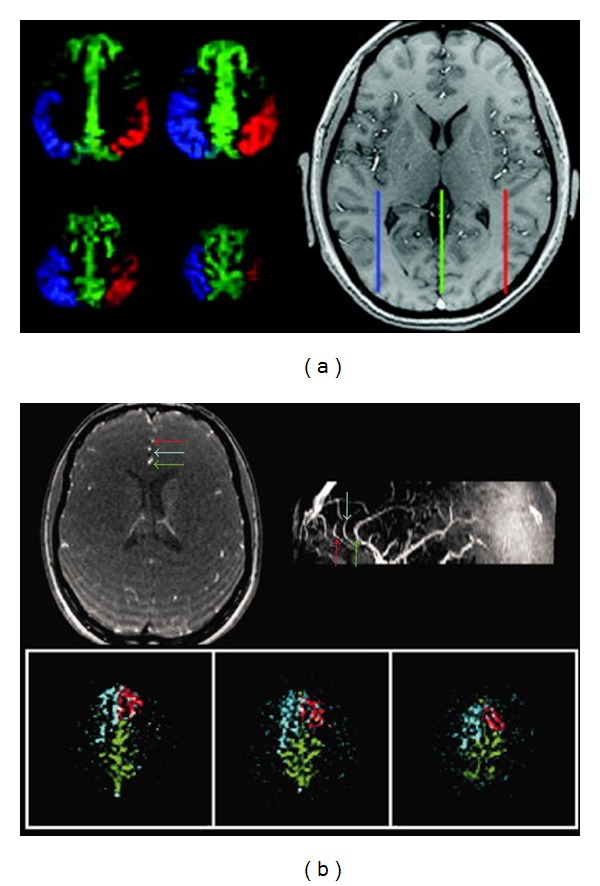
(a) Three-vessel encoding of vasculature above the Circle of Willis. In the labeling plane shown on the right, the ACA is well confined to the midline with the corresponding flow territory represented in pCASL perfusion maps as green. The territories supplied by the insular branches of the MCAs are also well labeled (shown in red and blue). Figure taken without modification from Wong [25]. (b) Selective encoding of three branches of the ACA using super-selective pCASL [26]. Top row shows TOF image and the corresponding saggital maximum intensity projection with the branches of the ACA color coded. The bottom row shows perfusion-weighted images of the territories fed by each vessel. Figure was taken without modification from Helle et al. [26].

Other modifications of the pCASL sequence that seek to selectively label individual vessels have been introduced by Wong [25] and Helle et al. [26]. Wong combined vascular territory imaging [27–29] with the pCASL sequence [22] to simultaneously acquire perfusion images of two or more vascular territories with SNR close to that of standard ASL within the same total scan time (Figure 2(a)). Applying a different sequence modification, Helle et al. also used pCASL to selectively and independently encode vessels feeding into different perfusion territories (Figure 2(b)). As we discuss later in this review, these new developments in ASL that allow for independent labeling of different flow territories are especially relevant for clinical research.

### 2.3. Pulsed ASL (PASL)

In contrast to CASL, labeling in PASL is achieved using more easily implemented short pulses (usually 10–15 ms) that invert spins in a specific region commonly referred to as the inversion slab (for a thorough review of PASL techniques cf. [5]). Depending on how labeling is applied with respect to the imaging volume, PASL techniques are divided into two main groups: symmetrical and asymmetrical. The original symmetrical PASL, called flow-sensitive alternating inversion recovery (FAIR), was developed in mid 1990s by Kwong et al. [30], Kim [31], and Schwarzbauer et al. [32]. The sequence consisted of two inversion recovery acquisitions: one involved slice-selective inversion, that is, the magnetization was inverted only at a selected slice, whereas the other was a non-slice-selective inversion. A delay was introduced after each inversion pulse and before image acquisition. After the delay, the tissue magnetization of the imaged volume of the slice-selective inversion includes the signal from the inflow of the uninverted blood. On the other hand, the tissue magnetization after the nonselective inversion is expected to be approximately equal to that of the tissue (assuming *T*_1_ of the blood to be approximately the same as the tissue *T*_1_, an assumption that works relatively well for gray matter). Similar to CASL, the difference between two consecutive images acquired using the two inversion types, respectively, results in a perfusion-weighted image. Although various versions of FAIR have been developed [5], the original FAIR sequence is still the most commonly used. Kim et al. extended the application of the original FAIR to multislice acquisition using a single inversion pulse to keep the temporal resolution sufficiently low for detecting motor activation [33]. Although FAIR is easy to implement and relatively straightforward in its application, multislice application remains problematic due to artifacts caused by imperfections in the profile of the inversion slice [5, 33].

The asymmetrical PASL sequence, called echo planar imaging and signal targeting with alternating radiofrequency (EPISTAR), was first proposed by Edelman et al. in 1998 [34, 35]. In this sequence, magnetization is inverted in a thick slab proximal to the imaging slice, followed by fast imaging (EPI) after a short delay that allows for the inverted magnetization to reach the imaged slice. The additional control image is similarly acquired after inverting magnetization in a slab symmetrically distal to the imaging slice and thus having the same MT effects. Proximal inversion with a control for off-resonance effects (PICOREs) [36] and transfer insensitive labeling technique (TILT) [37] sequences are based on the original EPISTAR technique.

As mentioned in the previous section, several ASL methods have been developed that, rather than labeling all of the feeding arteries, allow for selective imaging of individual perfusion territories. Regional perfusion imaging (RPI) technique developed by Van Laar et al. is based on concatenated TILT sequence labeling pulses and allows a labeling slab to be positioned at any angulation with respect to the imaging volume [38]. The RPI technique was the first to allow regional CBF measurement of individual feeding arteries. However, the sequence is very sensitive to magnetic field inhomogeneities and thus not best suited for high field imaging. A more recent sequence, pulsed star labeling of arterial regions (PULSARs), [29, 39] also based on the original EPISTAR sequence, has used an optimized water suppression pulse that presaturates the imaging volume thus increasing the sensitivity of the signal to flow [39]. Compared to the original RPI method, the PULSAR technique is less sensitive to field inhomogeneities, has better labeling efficiency and higher SNR. However, the sequences are difficult to implement and suffer from low sensitivity.

Combining the PULSAR labeling technique with a look-locker method for sampling at multiple time points and a periodic saturation scheme for clear definition of the arterial blood bolus, a quantitative STAR labeling of arterial regions (QUASAR) [40] technique was developed. Deconvolving the signal from multiple time points, the QUASAR method yields a simultaneous measurement of both arterial blood volume (aBV) and CBF [40].

Although it has yet to become routine in functional imaging of the brain, several other studies have reported on simultaneous measurement of aBV and CBF [3, 41, 42]. Simultaneous measurement of aBV and CBF using endogenous tracers may become an important tool in studying diseases in which the two physiological parameters may be dissociated.

### 2.4. Velocity Selective ASL (VSASL)

While the pulse sequence of VSASL contains all the main elements of the conventional ASL acquisition of label and control images, the difference is that in VSASL labeling is achieved based on the velocity of the arterial water rather than its position. Using velocity selective pulses, a velocity cutoff, *V*_*c*_, is imposed with the resulting labeled image containing, at least theoretically, only the spins whose velocity, *V*, meets the condition *V* ≤ *V*_*c*_. Assuming that the velocities in the arterial tree are monotonically decreasing, the amount of labeled blood in a given imaged voxel, that is, the ASL signal, is simply

(2) $${\text{ASL}}_{\text{signal}}=\text{PLD}\cdot \text{CBF},$$

where CBF and PLD represent the amount of flow and the postlabeling delay for that voxel [9].

An implication of (2) is the interdependence of the ASL signal on *V*_c_ via CBF and PLD. Wu et al. performed a systematic evaluation of the interaction between PLD and *V*_c_ and showed that while the experimental data were in good agreement with the expected flow values in gray matter, a significant signal from large vessels persisted for velocities up to *V*_c_ = 4 cm/s [43]. Therefore, the authors recommended a low cutoff *V*_c_ = 4 cm/s for quantitative measurement of tissue perfusion.

## 3. Which ASL Is Better?

With all the available ASL implementations, the obvious question is “which one is the best”? The answer is complex and perhaps warrants a review paper of its own, but the choice would depend on the application and should obviously involve, among others, considerations of availability of hardware, software, and technical expertise, as well as brain coverage needed and SNR assumed by the power analysis for the tested hypotheses. Unfortunately, the choice is often based on availability rather than scientific considerations. The need for technical expertise and sequence development, which to a certain degree depend on the type of scanner available, has hampered application-specific optimization of ASL imaging.

Generally, PASL has been more widely used because it is easier to implement and conceptually more straightforward than CASL. Also, because shorter labeling pulses are needed, PASL sequences are less affected by MT than the standard CASL sequence. However, drawbacks are still present such as low SNR, high sensitivity to transit times, and slice profile artifacts that can limit brain coverage. Although more difficult to implement, CASL, on the other hand, has been shown to yield higher SNR for whole brain imaging than PASL. While there is promise in VSASL, the technique is still relatively new, and more studies are needed to assess its sensitivity and applicability in disease and activation studies [44].

The recent development of pCASL, which draws on the respective advantages of CASL and PASL to provide reliable perfusion images with high SNR, has contributed to a substantial increase in applications of ASL at 3T. Due to its high efficiency, multislice capability, and relative ease of implementation without over-taxing the hardware, pCASL is becoming the best choice for a broad range of applications in brain research. Also, the potential of pCASL to selectively label vessels varying in size and orientation without compromising the SNR may prove invaluable in studying disease diagnosis, progression, and treatment.

With the concomitant advances in parallel imaging and fast acquisition pulses, ASL is primed to become the essential fMRI method for brain research. However, as mentioned above, the ASL signal is transformed into a physiological unit of CBF using a set of known or assumed MR and physiological parameters such as relaxation times, partition coefficient, transit times, inversion efficiency, and so forth [45]. It follows that any error in the estimation or assumption of these parameters would affect the absolute quantification of CBF. Furthermore, ASL images typically go through a processing algorithm that involves realignment, tissue segmentation, and normalization to the MNI or Talairach space for group analyses [46]. A detailed description of how each of these parameters and steps can affect quantification of flow is beyond the scope of this review. Here we focus on the basic principles of CBF quantification and describe recent analytical methods that have been developed to increase ASL's sensitivity for detecting changes in CBF while minimizing the effects of confounds such as partial volume effects (PVEs) and arterial transit times (ATTs).

## 4. From Signal to Absolute Quantification of CBF

ASL is based on the theory of tracer kinetics, which was first applied for measuring CBF in humans by Kety and Schmidt in 1948 [47]. In ASL, the tracer is the magnetically labeled arterial water, which is a diffusible endogenous tracer. The theory provides the mathematical tools that describe the relationship between the arterial concentration of the labeled water and the resulting tissue concentration. These mathematical tools were the basis of the “general kinetic model for quantitative perfusion imaging with ASL” developed by Buxton et al. [48] in which the Bloch's equation for longitudinal magnetization was modified to include delivery and clearance terms proportional to local blood flow as shown in (3):

(3) $$\frac{dM_{T}\left(t\right)}{dt}=\frac{M_{T}^{0}-M_{T}\left(t\right)}{T_{1}}+f\cdot \left(\frac{\lambda M_{A}\left(t\right)-M_{T}\left(t\right)}{\lambda }\right),$$

where *M*_*T*_^0^ is the equilibrium magnetization of tissue, *λ* is the partition coefficient for water, and *M*_*T*_ and *M*_*A*_ represent the time-dependent longitudinal magnetizations of tissue and arterial blood, respectively, [48].

Based on this generic model, several solutions have been suggested for both CASL and PASL techniques [39, 45]. Also, based on the timing of the acquisition parameters, models have been constructed to mathematically describe the signal from the various compartments within the brain [2, 21, 45]. For a short postlabeling delay, most of the label is assumed to be in the arterial compartment whereas for longer delays a two-compartment model separates the tissue signal from that of the arterial blood. Although numerous studies have shown good agreement of CBF values with the more conventional flow measurement techniques such as autoradiography, microsphere method, and PET [49–51], there are several confounds that affect the absolute quantification of CBF with ASL. In the next few sections, we review some of these confounds and describe new analytical methods that have been developed to minimize their impact on CBF quantification.

### 4.1. Partial Volume Effects

One of the constraints in ASL imaging is the need for fast image acquisition to ensure that the signal from the labeled blood is captured before it relaxes to its equilibrium state. Fast imaging is done at the expense of spatial resolution, which means that the signal from a given voxel will reflect a mixture of signals generated from all the three main brain tissues—gray matter (GM), white matter (WM), and CSF—comprising the voxel [52]. Because the flow values from each of these tissues are different, a difference in flow values between two voxels could be simply due to a difference in tissue heterogeneity rather than a true difference in flow. These cross-tissue contamination effects, known as partial volume effects (PVEs), are a direct consequence of limited spatial resolution in imaging in general. In ASL, PVEs are exacerbated by the nonlinear dependency of its signal on tissue heterogeneity via contributions from GM, WM, and CSF in the control image, *M*_*C*_, in the denominator of (1), (cf. [52] for details).

PVEs can be quite appreciable in cortical regions where GM can be as thin as 2 mm. To give a sense of the magnitude of these effects, a voxel containing 80% GM and 20% CSF would be generally assumed and analyzed as a GM voxel [52]. For such a voxel, a simple calculation based on (1) and assuming the relative tissue magnetization intensities for SE-EPI to be M_CSF_ : M_GM_ : M_WM_ ~1.6 : 1.2 : 1.0, CBF in GM would be underestimated by ~24% [52].

Recently, postprocessing algorithms have been developed to correct for PVEs in ASL imaging [52, 53]. In the original method developed by our group, a linear regression algorithm is spatially applied on the difference, (*M*_*C*_ − *M*_*L*_), image as well as the control, *M*_*C*_, image. The algorithm assumes that within a given spatially selective kernel, the equilibrium magnetization values and blood flow are uniformly distributed [52]. For the *M*_*C*_ image, the algorithm models the equilibrium magnetization of a given voxel as a weighted sum of contributions from each tissue and each voxel within the kernel. For the (*M*_*C*_ − *M*_*L*_) image, that is, the perfusion-weighted image, the intensity at each voxels is expressed as a weighted sum of flow distributions from GM and WM within the kernel, independently. In both cases, the weighting coefficients are the tissue's fractional volumes obtained as posterior probability values from the segmentation of a high-resolution images [52].

This method yields a measure of flow, referred to as “pure flow” or “flow density” that is independent of voxel's tissue content. In other words, one can compute a *pure* gray matter CBF (CBF_*d*_*GM*_) and a *pure* white matter CBF (CBF_*d*_*WM*_) image, independently. For each voxel, CBF_*d*_ from a given tissue represents the amount of blood flow the voxel would have were it comprised entirely of that tissue [52]. As we discuss in some detail below, this novel parameter, CBF_*d*_, has been shown to be more sensitive in detecting changes in CBF over time than the net CBF obtained with conventional ASL [54, 55].

**Figure 3:**
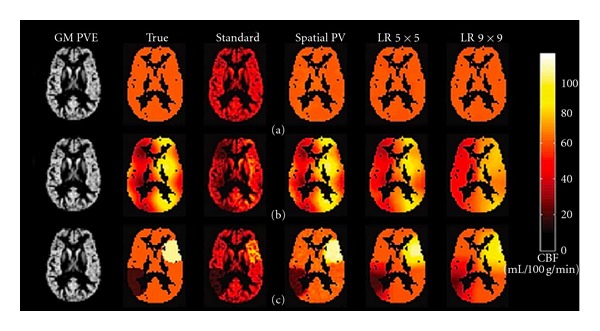
CBF images from three simulation data sets: (a) homogenous gray matter CBF, (b) superimposed spatially sinusoidal fluctuation, and (c) localized regions of hypo- and hyperperfusion. CBF images from conventional CASL (3rd column) are compared with those from PVE correction performed in the time domain [41] (4th column) [40], and PVE correction done spatially with a small and a large kernel size (columns 5th and 6th, resp.). Note that the time-domain PVE (4th column) retains the spatial features of the true hypo/hyperperfused regions (2nd column) whereas the spatially applied PVE method has a smoothing effect that increases with the size of the kernel. Figure was taken without modification from Chappell et al. [53].

A disadvantage of this method is that the linear regression is applied spatially thus causing an inherent spatial smoothing of the raw data. Due to SNR considerations, the larger the spatial kernel of the linear regression the higher the SNR. On the other hand, the larger the kernel, the larger the smoothing effect of the PVE correction (PVEc) algorithm. This could be detrimental to detecting localized changes in CBF such as those found in stroke or in highly localized activation paradigms. To circumvent this drawback, Chappell et al. have implemented the above method in the time domain by acquiring multiple ASL images with varying delay times [53]. Because it is applied in time rather than space, this PVEc method protects spatial features of CBF, thus avoiding the introduction of added smoothness to the boundaries of the regions of hypo- or hyperperfusion (Figure 3). In this case, the drawback is the time needed to acquire the data, which is an impediment especially for activation studies or studies where the patient's time in the scanner is restricted.

### 4.2. The Transit Times Confound

For all ASL techniques, blood is labeled at a location distal from the region of interest. Therefore, if acquisition were to take place immediately following the labeling pulse, not all of the labeled blood would have made it into the tissue, and consequently CBF would be misestimated; a voxel containing arterial blood with labeled spins destined for another voxel would have its CBF overestimated whereas a voxel that was imaged prior to all the labeled blood having reached it would have its CBF value underestimated. To describe the transit times of the blood from the labeling location to a given imaged voxel, two physiological parameters have been defined: arterial transit time (ATT), which represents the average time it takes the blood to cross the vasculature from the labeling plane to the microvasculature in the region of interest, and tissue transit time (TTT), which is the time it takes labeled blood to exchange with region's tissues [2]. The postlabeling delay is inserted at the end of the labeling pulse to allow for the labeled blood to reach the volume of interest and exchange with the tissue. However, a compromise needs to be made between the length of the delay and loss of signal due to relaxation processes. For regions with long transit times, the delay will still not be sufficient, and the interpretation of data becomes more complicated [2].

For certain applications where CBF needs to be measured at a localized region, the distance between the labeling location and the region of interest can be shortened (within the limits imposed by the MT effects) and the effect of transit times minimized. However, for many studies, hypotheses involve whole brain acquisition of the CBF in which the CASL techniques with labeling plane positioned in the carotid would be the method of choice. In this case, different areas of the brain will have different transit times therefore quantification would require knowledge or estimation of these times for each region. For studies in which the one-compartment arterial model is employed, that is, when the labeled blood is assumed to be mostly in macrovasculature, ATT is the main parameter that needs to be estimated; for the two-compartment model that includes the tissue microvasculature, estimation of TTT becomes essential.

By acquiring multiple ASL images at varying postlabeling delay values, ATT can be estimated via a parametric fit of the curves representing the fractional ASL signal versus time [2]. Because this step requires relatively long scanning times, ATT is not routinely measured in ASL imaging. Instead, when computing the CBF, ATT values are generally assumed to be either homogeneous throughout the brain or uniformly distributed within an acquisition slice and varying linearly with the ascending slice positions.

**Figure 4:**
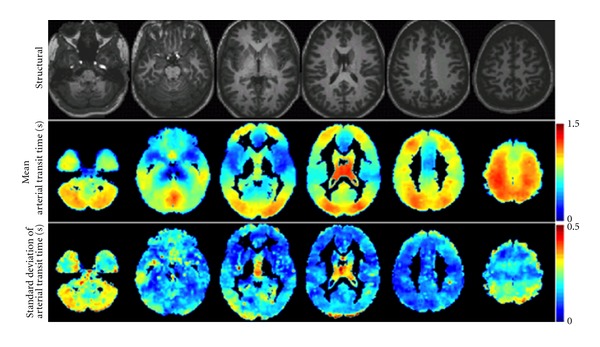
Voxelwise estimation of ATT values using a multiple-labeling-duration acquisition described in Borogovac et al. [55]. The units in the color bars are in seconds. Note regional heterogeneity in group mean ATT shown in the 2nd row. Also, across subjects standard deviation maps (3rd row) indicate variability in ATT especially in the posterior regions. This variability is expected to be higher in disease. Figure taken with permission from Borogovac et al. [55].

Recently, there have been two developments in estimation of ATT: first, improvements in technology and increased SNR at higher fields have allowed for voxelwise and ROI-wise parametric fitting of the multiple-PLD curves. Second, a method that varies labeling duration rather than postlabeling delay has been developed [55]. This method has been shown to be ~30% shorter than the multiple-PLD method [55]. Results have shown substantial heterogeneity in mean ATT values across the brain and across subjects even for healthy young volunteers (Figure 4), [55].

To estimate TTT, Wang et al. proposed a method that involved the use of flow encoding bipolar gradients to obtain the ratio of the perfusion signals in the vascular and microvascular compartments as a function of postlabeling delay [56]. The global mean tissue transit time was estimated at 1100 and 1400 ms for two conditions of the bipolar gradients with encoding velocity of 29 and 8 mm/sec, respectively. The mean TTT measured within cerebral vascular territories was shortest in the deep middle cerebral artery (MCA). As proof of concept, the method was applied on two patients with cerebrovascular disease where prolonged tissue transit times were demonstrated in the affected hemisphere [56]. However, the method suffers from dependency on the specific encoding velocity, and its routine application is hampered by the need for multiple-PLD acquisition.

With the above general background on the theoretical basics of ASL fMRI and its confounding factors in assessing CBF, we proceed with reviewing the advantages and some of the experimental challenges for applications of ASL in basic and clinical research.

## 5. ASL fMRI: Better Than BOLD?

Several imaging methods have been developed that exploit the neurovascular coupling of neuronal activity to local changes in CBF cerebral blood volume (CBV), and other physiological correlates [57]. The most pervasively used has been blood oxygenation level-dependent (BOLD) MRI, which, since its discovery in the early 1990s has been extensively used to map regions in the brain that respond to task-specific activation [58].

BOLD is a susceptibility-based method that creates “functional,” *T*2*-weighted images by exploiting local inhomogeneities in the magnetic field due to changes in the relative concentrations of oxygenated and deoxygenated hemoglobin (dHb) accompanying brain activation [58].

In contrast with the nuclear medicine methods, both BOLD and ASL MRI use endogenous tracers and therefore are completely noninvasive and more readily available. Because BOLD has higher SNR and higher temporal resolution than ASL, it is more suited for event-related designs, especially when absolute quantification is not essential to the hypothesis being tested. Also, BOLD is easier to implement and does not usually require any additional programming of the RF and gradient pulses already provided by the manufacturer.

However, ASL offers several advantages over BOLD, especially in applications where slow varying changes in brain function are investigated:

**(1) Spatial Localization**

Because the BOLD effect originates from an intricate interplay between changes in CBF, CBV and oxygen consumption, its signal is composite in nature and unable to pinpoint to a single correlate of neuronal activity [59–61]. Furthermore, because the signal comes primarily from the intravascular dHb, the spatial correlation to the actual site of activation is relatively poor with considerable spatial spreading onto the venous structures [62]. In contrast, the ASL signal is straightforward to interpret because it reflects, at least theoretically, a single physiological process, namely, CBF. Consequently, the task-specific patterns mapped with ASL yield better spatial correlations with the actual site of regional involvement than BOLD [63].

**(2) Signal Quantification**

The conclusions from BOLD studies have been mainly qualitative in nature as the baseline values are commonly unaccounted for, and the signal is typically expressed in percent change [64]. The effect of baseline variability in BOLD fMRI data has been experimentally shown by Cohen et al. [65] and Brown et al. [66]. Both studies reported a mismatch between the change in baseline CBF and the corresponding BOLD response on the same subjects and for the same stimulus [65, 66].

ASL, on the other hand, yields a physiologically quantifiable measure thus allowing baseline levels to be directly compared before and after activation [21].

**(3) Power Spectrum**

**Figure 5:**
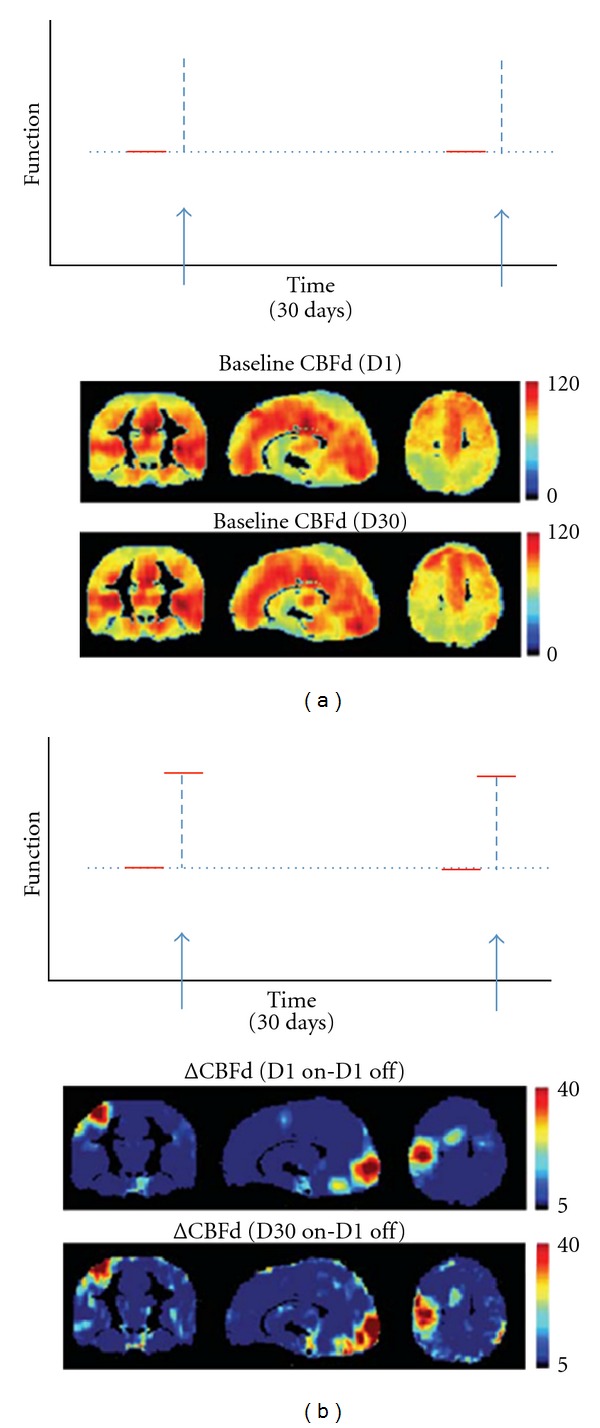
Tracking functional changes over one month. (a) Comparing baseline CBF on day 1 (indicated by first red horizontal line in upper panel, illustrating experimental design), and baseline CBF on day 30 (second red line in upper panel) shows stability over time. Middle panel shows the whole brain maps on day 1, and lower panel shows whole brain maps on day 30. (b) Comparing acute CBF changes induced by visual or motor stimulation on day 30 to baseline CBF on day 1 (as illustrated in the upper panel) is similar to acute changes induced by visual or motor stimulation on day 1 to baseline CBF on day 1. Middle panel shows the whole brain maps of day 1 stimulation to day 1 baseline, and lower panel shows whole brain maps of day 30 stimulation to day 1 baseline. Maps show similar motor and visual cortex activations. (Note that this is a modified version of Figure 3 in Borogovac et al. [55]).

The power spectrum of the BOLD signal shows higher amplitudes at low frequencies in what has been described as the 1/f noise. This temporal autocorrelation makes BOLD fMRI unsuitable for application in experimental designs with fundamental frequency below 0.01 Hz, that is, for task events spaced by more than ~90 seconds apart [67]. In contrast, due to pairwise subtraction of adjacent time points, the power spectrum of ASL is essentially frequency independent, which makes it ideal for tracking slow varying changes in the brain such as those due to emotional responses, mood changes, disease, drug therapy, and so forth. In a recent study, Borogovac et al. [55] used PVEc ASL fMRI to compare changes in CBF due to motor-visual activation within the same session and across two sessions separated by 1 month (Figure 5). The study underscored the utility of the CBF_*d*_ parameter mentioned above in detecting longitudinal changes in CBF. Because this physiological parameter is relatively independent of tissue heterogeneity across subjects, it was more stable across time and ~60% more sensitive in detecting changes due to activation [55].

**(4) Susceptibility Effects**

Because BOLD is a susceptibility-based technique, gradient echo (GE) EPI is commonly employed to achieve maximum sensitivity. Consequently, BOLD is prone to artifacts in the areas with high susceptibility such as those around tissue-bone or tissue air boundaries, especially at high fields. ASL, on the other hand, can be combined with spin echo (SE) imaging to reduce bulk susceptibility artifacts thus yielding greater sensitivity in lower brain regions and more precise localization [68]. However, as higher field scanners become more available, the feasibility of using spin-echo-based BOLD fMRI is also increasing.

## 6. ASL fMRI in Aging and Disease

The BOLD response is a sensitive indicator of *where* neural activity occurs, but it is very difficult to interpret the magnitude of the BOLD response as a quantitative reflection of underlying physiology. The effect of the baseline state is perhaps the most serious issue for interpreting BOLD measurements in disease. For example, in a recent study of subjects at risk for AD, Fleisher and colleagues have found a reduced BOLD response in the hippocampus to a memory task in the at-risk subjects compared with controls [69]. However, by including ASL measurements as well, they found that, during the performance of the task, the two groups had similar absolute levels of flow, but that flow in the baseline state was elevated in the at-risk group [69].

According to a 2008 review paper by Deibler et al. [70], during a period of one year more than 3000 ASL procedures were performed as part of routine clinical brain MRI evaluation at 1.5 T and 3.0 T. As mentioned above, much of the value of ASL imaging comes from its noninvasive nature and the fact that it can be acquired within a routine MR scan commonly prescribed to patients.

In general, ASL applications in the clinical realm can be divided into two main groups: vascular diseases such as stroke and carotid occlusive diseases [71–73], and “functional” diseases, including normal aging [54], Alzheimer's disease (AD) [74], and schizophrenia [75]. This dichotomization is not meant to be inclusive, but it serves to underscore the physiological basis for the observed CBF measure. In vascular diseases, changes in CBF are to a response in structural changes in the brain, such as carotid occlusions, hematomas, tumors, or the advent of a stroke and other ischemic events. In “functional” diseases, changes in CBF (in time or as compared to healthy populations) can occur independently of structural changes in the brain or precede them.

In their recent review of ASL applications in routine clinical practice, Deibler et al. described the use of ASL for a range of diseases where hyperperfusion can be detected both focally, as in luxury perfusion, spontaneous recanalization, seizure activity, tumors, among others, and globally, as in young populations, or during conditions of hypercapnia, and reported cases of postcarotid endarterectomy [70, 76].

It is important to emphasize that the ASL confounds described in the section above become even more relevant in clinical applications. For example, in studies of stroke and carotid occlusive diseases, estimation of transit times is of primary importance [71, 72]. In this case, concomitant measurement of CBF would increase the reliability of the transit time measurements and would make the interpretation of the results more straightforward.

In studies that involve comparison of CBF between young and elderly populations, PVE becomes a main confound because of the atrophy present in the latter [54]. Recently, our group has applied the PVEc algorithm to ASL data acquired on young and elderly populations. The largest PVE contribution was found in the frontal lobe and accounted for an additional 10% and 12% increase in the age-related CBF difference between men and women, respectively, [54].

## 7. Future Directions in ASL fMRI

There are two significant challenges in ASL imaging that continue to hamper its routine application in brain research and comprise the main focus of current ASL development research: low SNR and relatively low temporal resolution. Our numerical simulations, based on tissue relaxation times at 3 T, assuming average GM CBF_d_ of 100 mL/(100 g × min) [77] and Gaussian distribution of noise, have shown that the highest achievable SNR is ~4%. High field imaging is beneficial for ASL because, in addition to the expected increase in the SNR due to field considerations, there is an increase in SNR that is due to longer T1 values at higher fields; increased T1 translates into less labeling loss, that is, signal loss, due to relaxation. Wang et al. showed that, for PASL, the SNR and CNR increased 2.3x and 2.7x, respectively, for resting state perfusion at 4 T compared to 1.5 T [21]. However, there was no significant improvement in sensitivity for detecting changes in CBF due to motor activation [21], which the authors attributed to increased physiological noise and susceptibility-related artifacts at 4 T. Because of the need for fast sequential scanning of control and labeled images, ASL has generally relied on EPI imaging, which is problematic in higher fields due to field homogeneity imperfections that introduce distortions in regions of high magnetic susceptibility. One solution has been to combine fast three-dimensional (3D) sequences with ASL imaging to provide higher SNR while reducing image distortions [24, 78]. Another approach for increasing SNR in ASL is the use of a phase array receiver coils, which allow for image acquisition with shorter echo times; a decrease in echo time is beneficial both in terms of SNR and in reducing distortions due to susceptibility artifacts [79].

As mentioned above, temporal resolution is also inherently poor in ASL, especially for detecting fast changes in brain function due to activation. This is a direct consequence of the pairwise acquisition in ASL; to obtain one CBF image, two images, control and label, have to be acquired, thus doubling the effective TR, which generally varies between 4 s to 8 s. So far there have been two emerging methods for improving temporal resolution in ASL: turbo-ASL [9] and single-shot ASL [80]. Because of the complexity of signal quantification in both techniques, they are restricted to applications in which absolute quantification is not of primary importance. Hernandez-Garcia et al. combined a two-coil approach with turbo CASL for detecting perfusion responses in both block-design and event-related experiments [81]. The higher temporal resolution was achieved by collecting the control and labeled images after a single labeling period. With the advantage of optimum SNR of CASL and increased temporal resolution, the authors reported satisfactory sensitivity for detecting perfusion response to an event-related paradigm [81].

It has become clear that, given the complexity of questions in brain research today, no single technique can be the panacea of the experimental challenges we face in answering them. The solution relies on combining the advantages of various imaging techniques with advances in analytical methods for better evaluation of the physiological parameters that underlie brain function at various states. In this regard, ASL development is branched in three directions: first, developing new implementations of the technique that are more suited to applications in higher fields and can increase the spatial and temporal resolution of CBF imaging. At the moment, pCASL combined with novel fast imaging sequences holds the best promise.

The second is combining baseline ASL CBF measurement with BOLD, in what is known as calibrated BOLD fMRI [61]. With a separate measurement of blood flow with ASL, it is possible to calculate how much the oxygen metabolism would have to change to give the measured BOLD response [82]. However, until an alternative is found to the requirement for measurement under hypercapnic condition, calibrated BOLD has yet to find wide application in the clinical realm.

Third, because ASL suffers from low SNR, advances in analytical methods that boost the sensitivity of the method are imperative. Perhaps more important, sophisticated analytical methods allow us to ask more sophisticated questions about brain function. For example, recently ASL fMRI has been combined with multivariate analysis to detect covariate CBF patterns that could distinguish AD patients from healthy controls with 95% specificity and 100% sensitivity [83].

These recent insights and technical developments suggest that ASL fMRI is on the cusp of realizing its full potential for brain research.
