# Erratum to “Arterial Spin Labeling (ASL) fMRI: Advantages, Theoretical Constrains and Experimental Challenges in Neurosciences”

**DOI:** 10.1155/2012/658101

**Published:** 2012-11-11

**Authors:** Ajna Borogovac, Iris Asllani

**Affiliations:** Department of Radiology, Columbia University, New York, NY 10032, USA

The old title in the original paper was “*Arterial Spin Labeling (ASL) fMRI: Advantages, Theoretical Constrains and Experimental Challenges in Neurosciences*” and it will be changed to “*Arterial Spin Labeling (ASL) fMRI: Advantages, Theoretical Constraints, and Experimental Challenges in Neurosciences*.”

